# Influence of the detection of parent-of-origin on the pregnancy outcomes of fetuses with copy number variation of unknown significance

**DOI:** 10.1038/s41598-020-65904-2

**Published:** 2020-06-01

**Authors:** Lin Chen, Li Wang, Daishu Yin, Yang Zeng, Feng Tang, Jing Wang

**Affiliations:** 1 Department of Obstetrics and Gynecology, West China Second University Hospital of Sichuan University, Chengdu, 610041 China; 2 Key Laboratory of Birth Defects and Related Diseases of Women and Children, Ministry of Education, Sichuan University, Chengdu, 610041 China

**Keywords:** Genetics, Molecular biology

## Abstract

The widespread application of high-resolution chromosome detection technology in clinical practice has identified many variants of unknown significance (VOUS) in prenatal diagnosis. The purpose of this study was to prospectively analyze the chromosomal results of parents and the follow-up information of pregnancy outcomes of prenatal samples with VOUS, so as to determine the influence of the detection of parent-of-origin on the pregnancy outcomes of fetuses with VOUS. The present study analyzed amniotic fluid samples obtained from women with different risk indications between February 2017 and December 2018. The samples were subjected to copy number variation sequencing, and detection of parent-of-origin was suggested in cases of samples with VOUS. The pregnancy outcome was followed up. In a total of 14073 amniotic fluid samples, 729 cases of VOUS were detected (5.2%, 729/14073) and 721 cases were followed up successfully. Among the 721 cases, 525 patients agreed to detect the parent-of-origin (72.8%, 525/721). It was revealed that the VOUS in 460 of the fetuses were hereditary (87.6%, 460/525). The percentages of abnormal pregnancy outcomes (included pregnancy loss, fetal pathological abnormality, preterm delivery, neonatal death, birth defects) in the inherited, de novo, and refusal to detect the parent-of-origin (i.e. unknown origin) groups were 4.3% (20/460), 6.2% (4/65), and 6.6% (13/196), respectively. There was no significant difference among the three groups (P > 0.05). The rate of voluntary termination of pregnancy (TOP) in the unknown origin group was significantly higher than that in the group that had determined the parent-of-origin (14.3% vs 7.4%, P = 0.005). There is currently no evidence that suggests that the proportion of abnormal pregnancy outcomes is higher in fetuses with VOUS than in other fetuses. However, the present study revealed that determining the parent-of-origin affects the decision to undergo voluntary TOP, as the rate of voluntary TOP in the group that refused detection was higher than that in the group that consented.

## Introduction

Since its introduction in 1970, amniocentesis remains the most commonly used invasive prenatal diagnostic tool; it is primarily used for the prenatal diagnosis of fetal chromosomal diseases, single gene diseases, and congenital metabolic diseases^1,2^. The incidence of birth defects in China is ~5.6%^3^, and chromosomal aberrations account for >80% of the genetic causes of birth defects^4^. At present, >300 chromosomal microdeletions or microduplication syndromes caused by pathogenic copy number variation (CNV)^5,6^ have been described, and the combined incidence rate is nearly 1/600^5^, accounting for half of the birth defects caused by chromosomal aberrations^4^.

Chromosomal microarray analysis (CMA) is a classical method used to detect CNVs in the genome, particularly abnormal submicrostructures. The advent of affordable and rapid next-generation sequencing has transformed prenatal diagnosis. At present, high-resolution chromosome detection techniques such as CMA and copy number variation sequencing (CNVseq) are widely used in fetal chromosome detection in China^7–12^. The application of CMA and CNVseq in the prenatal population provides additional and clinically significant cytogenetic information compared with conventional karyotyping. CMA and CNVseq have an additional detection rate of 1–6% for clinically significant CNVs^7,13–17^ and may also identify variants of unknown significance (VOUS), with a detection rate of 1.4–3.4%^14,16,17^. VOUS refers to the genomic variation that cannot be accurately explained based on the current understanding of the human genome and available databases. Therefore, it may be necessary to investigate the parents’ genome and perform a comprehensive analysis of the family, to aid the interpretation of the fetal test results^18^.

In this study, we prospectively analyzed the results of parent-of-origin detection and the follow-up data of pregnancy outcome of fetuses with VOUS, as detected by CNVseq technology. The results may shed light on the influence of the detection of parent-of-origin on the pregnancy outcomes of these fetuses and provide practical suggestions for prenatal fetal chromosome detection schemes and genetic counseling.

## Results

In total, 14073 amniotic fluid samples were analyzed between February 2017 and December 2018, and 729 cases (5.2%) of VOUS were detected. Among these, 8 cases were lost to follow-up and 721 cases were included in this study. The average age of the pregnant women was 31.1 years (range, 17–44 years), and the average gestational age at amniocentesis was 22.4 weeks (range, 18.1–35 weeks).

The parents of 525 fetuses with VOUS agreed to detect the parent-of-origin (72.8%, 525/721). It was revealed that 87.6% (460/525) of the fetuses inherited the CNV from the father or mother; in total, 51.0% were maternal, 34.9% were paternal, 1.7% were parental and 12.4% were de novo. The parents of 196 fetuses (27.2%) refused to detect the parent-of-origin, mainly owing to too worried about the adverse risks of VOUS to the fetuses and late pregnancy. After excluding the presence of VOUS in the sex chromosome (113 cases), 608 cases were identified in the autosomal chromosomes. Among the 608 cases of autosomal VOUS, 445 cases agreed to detect the parent-of-origin, and maternal, paternal, parental, and de novo VOUS accounted for 49.0, 35.5, 1.6, and 13.9%, respectively. In total, 163 cases (26.8%) refused to detect the parent-of-origin. The results showed that VOUS in the sex chromosomes had no significant effect on the parents’ decision to detect the parent-of-origin. In addition, the characteristics (including duplication or deletion, fragment size) of fetal CNV in the consented to detection of parent-of-origin group and the rejection group were compared, there was no statistical difference between the two groups (P > 0.05), as shown in Table 1. We have made statistical analysis on the reasons for performing amniocentesis in the consented to detection of parent-of-origin group and the rejection group, and there was no statistical difference between the two groups (P > 0.05).

**Table 1:** The fetal CNV characteristics of consented to detection of parent-of-origin group and rejection group. CNV: Copy number variation.

CNV	Detection of parent-of-origin, n (%)		Total samples
	The consent group	The rejection group	
**Deletion or duplication of CNV**			
Deletion	144 (27.4%)	59 (30.1%)	203 (28.2%)
Duplication	371 (70.7%)	133 (67.9%)	504 (69.9%)
Deletion with duplication	10 (1.9%)	4 (2.0%)	14 (1.9%)
**Size of CNV**			
<1 Mb	262 (49.9%)	104 (53.1%)	366 (50.8%)
1–2 Mb	213 (40.6%)	68 (34.7%)	281 (39.0%)
>2 Mb	50 (9.5%)	24 (12.2%)	74 (10.2%)
Total	525 (72.8%)	196 (27.2%)	721 (100%)

In the present study, abnormal pregnancy outcomes included preterm delivery, developmental delay, hemangioma, hearing impairment, congenital heart disease, brain tumors, eversion of the umbilicus, polydactyly, neonatal death, termination of pregnancy (TOP) due to fetal pathological factors (ultrasound-detected structural abnormalities and thalassemia), and pregnancy loss. The rate of abnormal pregnancy outcomes was 4.3% (20/460) in the inherited group, 6.2% (4/65) in de novo group, and 6.6% (13/196) in the unknown origin group. There was no significant difference among the groups (Yates’ continuity correction of the Chi-squared test and Pearson’s Chi-squared test, P > 0.05). The rate of voluntary TOP was 7.4% (39/525) in the group that consented to the detection of parent-of-origin (i.e. the sum of the inherited and de novo groups) and 14.3% (28/196) in the rejection group (i.e. unknown origin group). The rate in the latter group was significantly higher than that of the former (Pearson’s Chi-squared test, X^2^ = 7.961, P = 0.005). The pregnancy outcomes of the 721 cases with VOUS are presented in Table 2.

**Table 2:** The origin and pregnancy outcomes of the 721 cases with VOUS. TOP: Termination of pregnancy.

Origin	Outcome of pregnancy, n (%)					Total
	Normal infants	Abnormal infants	TOP (fetal pathological factors)	Pregnancy loss	Voluntary TOP	
Total samples	617 (85.6%)	19 (2.6%)	12 (1.7%)	6 (0.8%)	67 (9.3%)	721 (100%)
Inherited	432 (93.9%)	15 (3.3%)	4 (0.9%)	1 (0.2%)	8 (1.7%)	460 (63.8%)
Maternal	244 (91.0%)	12 (4.5%)	4 (1.5%)	1 (0.4%)	7 (2.6%)	268 (58.3%)
Paternal	179 (97.8%)	3 (1.6%)	0 (0)	0 (0)	1 (0.6%)	183 (39.8%)
Parental	9 (100%)	0 (0)	0 (0)	0 (0)	0 (0)	9 (1.9%)
De novo	30 (46.2%)	1 (1.5%)	2 (3.1%)	1 (1.5%)	31 (47.7%)	65 (9.0%)
Unknown origin	155 (79.1%)	3 (1.5%)	6 (3.1%)	4 (2.0%)	28 (14.3%)	196 (27.2%)

## Discussion

In total, 14073 amniotic fluid samples were analyzed in the present study, and 729 samples (5.2%) with VOUS were identified. The parents of the affected fetuses underwent CNVseq, and the pathogenicity of CNV was analyzed again. The VOUS ratio decreased to 1.9% (265/14073), which was similar to the VOUS ratio reported in the literature^14,16,17^. Of the 525 samples that agreed to detect the parent-of-origin, the vast majority (87.6%, 460/525) of the fetuses inherited the CNV from the father or mother without abnormal phenotype, the pathogenicity of CNVs in these samples should be changed to likely benign. Among the 721 cases with follow-up results, the proportion of parents who refused to detect the parent-of-origin was 27.2%, mainly owing to too worried about the adverse risks of VOUS to the fetuses and late pregnancy. In this study, amniocentesis was performed on some samples when they were >25th gestational week, the reason for chromosome examination of these samples was abnormal fetal ultrasound findings. At present, the ultrasound screening of fetal malformations in our country is mostly performed at 22–24 gestational weeks, so most of the fetal ultrasound abnormalities are usually found at this time. In addition, due to the large population of our country and few hospitals with amniocentesis qualification in our province, pregnant women usually need to line up for amniocentesis, so they cannot receive surgery in time. Therefore, we hypothesized that if the parent-of-origin was detected in all the samples, the actual proportion of VOUS samples may be lower.

In this study, abnormal pregnancy outcomes included preterm delivery, developmental delay, hemangioma, hearing impairment, congenital heart disease, brain tumors, eversion of the umbilicus, polydactyly, neonatal death, TOP due to fetal pathological factors (ultrasound-detected structural abnormality and thalassemia), and pregnancy loss. At present, a direct association between abnormal pregnancy outcomes and CNVs carried by the fetus cannot be established. The incidence of birth defects in China is ~5.6%^3^. It has been estimated that 7.9 million children are born with a serious birth defect of genetic or partially genetic origin annually, accounting for 6% of the total number of births worldwide^19^. The results obtained in the present study showed that the rate of abnormal pregnancy outcome was 4.3% in the inherited group, 6.2% in the de novo group and 6.6% in the unknown origin group. There was no significant difference among the groups, and there was no other evidence to suggest that the risk of abnormal pregnancy outcomes in samples with VOUS were higher than those in other samples. According to the follow-up results, the proportion of pregnancy loss or fetal death in the 721 samples was 0.8% (6/721). A Danish randomized controlled trial showed that the rate of fetal loss was 1.7% in the amniocentesis group and 0.7% in the control group, with a 1.0% procedure-related net risk^20^. A meta-analysis revealed that the weighted pooled procedure-related risk of miscarriage for amniocentesis was 0.11%^21^. A previously published study investigating 147987 invasive procedures in Denmark reported that the rate of miscarriage within 28 days after amniocentesis was 0.56% and that the risk of stillbirth within 42 days was 0.09%^22^. Compared with the aforementioned studies, VOUS does not seem to increase the risk of pregnancy loss or fetal death after amniocentesis. However, certain patients in the present study chose to undergo a TOP without detection of the parent-of-origin. The proportion of pregnant women choosing to terminate pregnancy voluntarily in de novo group was very high (31/65, 47.7%), so we were unable to investigate pregnancy outcomes in these cases. In addition, with the completion of the human genome map^23^, a large number of VOUS may be related to several clinical phenotypes^24^. Therefore, in fetuses with VOUS, it is recommended that clinicians increase patient monitoring, via examination and ultrasounds, and perform timely interventions and intrauterine treatment if necessary. Additionally, more intensive follow-up is required after birth.

The present study revealed that there was no statistical difference in the proportion of abnormal pregnancy outcomes among the inherited, de novo and unknown origin groups. The rate of voluntary TOP was 7.4% among patients who consented to detect the parent-of-origin, and 14.3% in patients who refused this approach. This difference was statistically significant (P = 0.005). The samples included in the “voluntary TOP” group refer to the samples without any other pathogenic abnormality (including ultrasound abnormality) except the CNV before pregnant women choose TOP. There was no significant difference in CNV characteristics and genetic counseling among groups, and no other factors that might lead to different pregnancy outcomes were found. The proportion of normal infants in the group of patients who consented to detect the parent-of-origin was 88.6% (462/525). Theoretically, the fetal outcome of the rejection group should be similar to that of consent group. However, compared with women who detected the parent-of-origin, a higher proportion of women who did not detect the parent-of-origin chose to voluntarily terminate their pregnancy, resulting in a lower proportion (79.1%, 155/196) of normal infants in the rejection group (462/525 vs 155/196, P = 0.001), as healthy fetuses may have also been terminated.

In conclusion, there is currently no evidence that suggests that the pregnancy outcomes of women with VOUS samples are worse than those of general pregnant women. Instead, parents with VOUS samples should detect the parent-of-origin in a timely manner to further establish the pathogenicity of VOUS. Economic resources permitting, we can learn from the detection strategy of whole exome sequencing in the field of identifying pathogenic genes of children’s rare diseases^25–27^, women who perform fetal chromosomal detection in late pregnancy should consider carrying out CNVseq of parent-child trios (trio-CNVseq). This approach may not only shorten the time to obtain the test report but may also produce more clear amniotic fluid chromosomal results, which can help relieve the anxiety of pregnant women and assist in the selection of an appropriate treatment plan. However, this proposal requires further analysis to comprehensively evaluate the health benefits and economic feasibility.

## Materials and Methods

### Biological samples

This study investigated amniotic fluid samples obtained from women with different risk indications in the West China Second University Hospital of Sichuan University between February 2017 and December 2018. Risks included advanced maternal age, increased risk of a screening test, abnormal ultrasound findings, obstetric history (previous fetus or child affected by chromosomal diseases), family history (parental carrier of chromosomal balanced translocation or inversion, parental chromosomal diseases or mosaicism for chromosomal diseases) or maternal request. The study was approved by the Medical Ethics Committee of West China Second University Hospital of Sichuan University, and all experiments were performed in accordance with relevant guidelines and regulations. All pregnant women received genetic counseling and signed informed consent.

### Amniotic fluid sample collection and DNA extraction

According to routine operation specifications, 20 ml amniotic fluid was extracted and placed into four sterile centrifuge tubes. CNVseq and quantitative fluorescence PCR (QF-PCR) were performed on two tubes, and the remaining two tubes were stored at 4 °C. DNA was extracted from the amniotic fluid using a DNeasy Blood and Tissue Kit (QIAGEN, Germany), according to the manufacturer’s instructions. QF-PCR detection was performed using 21 trisomy/sex chromosome/polyploid and 18 trisomy/13 trisomy/polyploid detection kits (DAAN Gene, China), according to the manufacturer’s instructions. If the results of QF-PCR indicated that there were maternal cells in the samples, CNVseq and QF-PCR were performed on the spare samples after cell culture.

### CNVseq

The DNA library was obtained using the Chromosome CNV Detection kit (Berry Genomics, China) and was subsequently sequenced on the Illumina Nextseq 500 sequencing platform (Illumina, United States) using the Nextseq 500 High Output kit (Illumina, United States). Finally, we compared the reads obtained by sequencing with the human reference genome and performed bioinformatics analysis to obtain the genomic copy number information of the samples as previously described^14^. In this study, the pathogenicity of CNVs >100 kb was analyzed. The clinical significance of the CNVs was interpreted according to the standards and guidelines for the interpretation of sequence variants recommended by the American College of Medical Genetics and Genomics and the Association for Molecular Pathology^18^. By searching the DGV (http://dgv.tcag.ca/), Decipher (https://decipher.sanger.ac.uk/), Clingen (https://www.ncbi.nlm.nih.gov/projects/dbvar/clingen/), and OMIM (http://omim.org/) databases and combining these data with fetal ultrasound results, we preliminarily classified the pathogenicity of CNVs into five categories: benign CNVs, likely benign CNVs, VOUS, likely pathogenic CNVs, and pathogenic CNVs.

### Detection of parent-of-origin

When VOUS were identified in the amniotic fluid samples, we recommended that the biological parents of the fetus underwent CNVseq (using peripheral blood samples) to determine the origin of the CNV of the fetus. The DNA extraction and CNVseq methods were performed as described for the amniotic fluid samples. Some samples had multiple CNVs at different genomic locations, if some of the CNVs in a sample were from the father and other CNVs were from the mother, the origin of the CNVs in the sample was “parental”. If the fetal CNV was inherited from the father or mother without abnormal phenotype, the pathogenicity of the CNV was likely benign. If the fetal CNV was De novo, the pathogenicity of the CNV was still VOUS.

### Follow-up of pregnancy outcome

**Figure 1:**
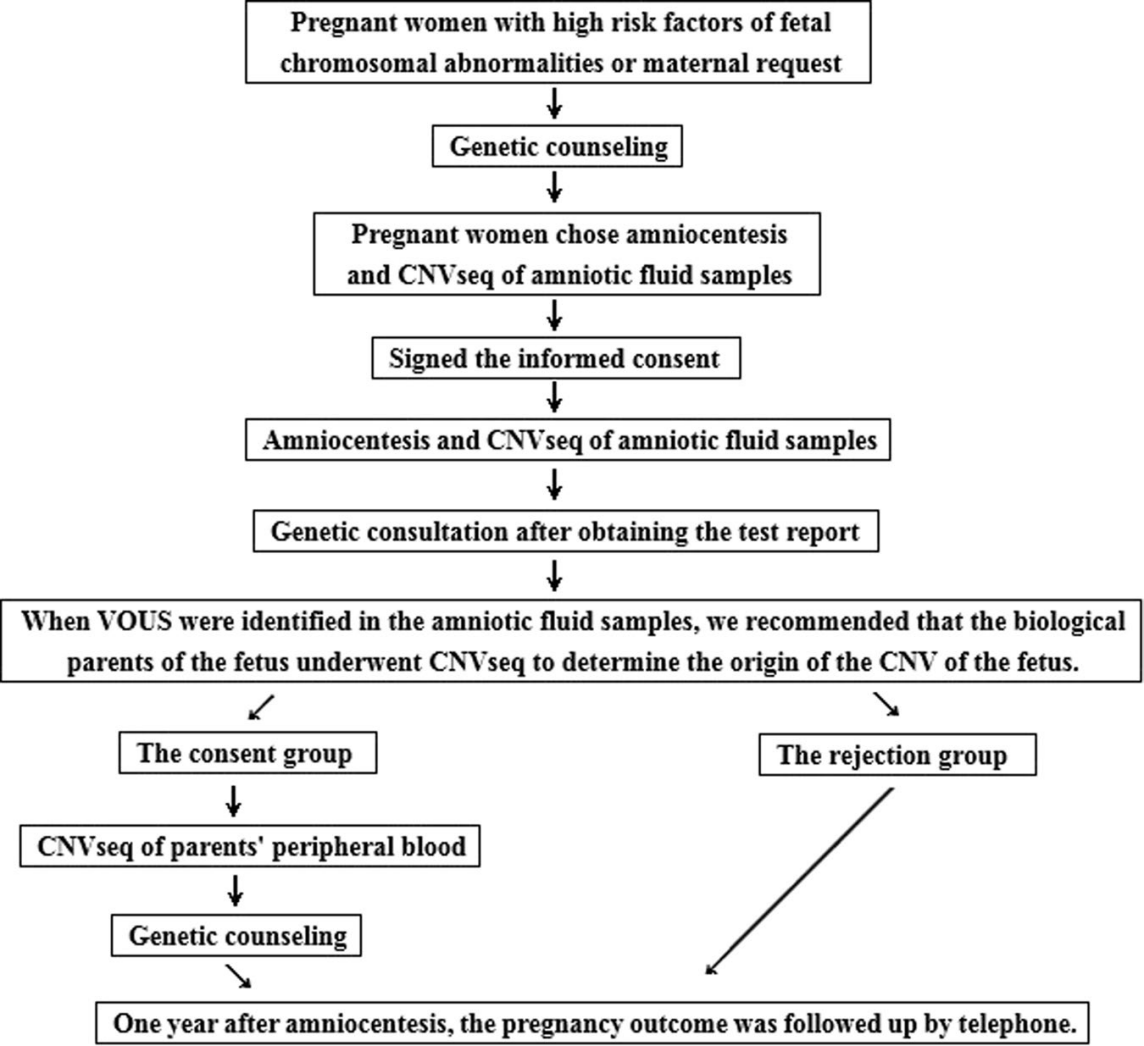
The flowchart of the study.

One year after amniocentesis, the mother or father of the fetus was contacted by telephone. The contents of the inquiry include: fetal ultrasound results during pregnancy, pregnancy complications of pregnant women, whether pregnancy loss, whether TOP and the causes, date of delivery, mode of delivery, weight and length of the newborn, the Apgar score, whether the appearance of the newborn was abnormal, feeding conditions after birth, examination results of pediatric outpatient service. The flowchart of the study is shown in Fig. 1.

### Statement

We confirm that informed consent was obtained from all participants and/or their legal guardians.

## References

[1] Sarto GE (1970). Prenatal diagnosis of genetic disorders by amniocentesis. Wis. Med. J..

[2] Ghi T (2016). International Society of Ultrasound in Obstetrics and Gynecology (ISUOG). ISUOG Practice Guidelines: invasive procedures for prenatal diagnosis. Ultrasound Obstet. Gynecol..

[3] Ministry of Health, People’s Republic of China. *Report on Prevention and Treatment of Birth Defects*. (2012).

[4] Evans MI, Wapner RJ, Berkowitz RL (2016). Noninvasive prenatal screening or advanced diagnostic testing: caveat emptorEJ3. Am. J. Obstet. Gynecol..

[5] Nevado J (2014). New microdeletion and microduplication syndromes: A comprehensive review. Genet. Mol. Biol..

[6] Weise A (2012). Microdeletion and microduplication syndromes. J. Histochem. Cytochem..

[7] Levy B, Wapner R (2018). Prenatal diagnosis by chromosomal microarray analysis. Fertil. Steril..

[8] Stosic M, Levy B, Wapner R (2018). The Use of Chromosomal Microarray Analysis in Prenatal Diagnosis. Obstet. Gynecol. Clin. North. Am..

[9] Cohen K (2015). Diagnosis of fetal submicroscopic chromosomal abnormalities in failed array CGH samples: copy number by sequencing as an alternative to microarrays for invasive fetal testing. Ultrasound Obstet. Gynecol..

[10] Zhu X (2016). Identification of copy number variations associated with congenital heart disease by chromosomal microarray analysis and next-generation sequencing. Prenat. Diagn..

[11] Collaboration group of the application of chromosome microarray analysis in prenatal diagnosis. (2014). Expert consensus on the application of chromosome microarray analysis in prenatal diagnosis. Chin. J. Obstet. Gynecol..

[12] Clinical genetics group, medical genetics branch, Chinese Medical Association; Prenatal diagnosis Committee of genetic diseases, branch of medical geneticists, Chinese Medical Doctor Association; Genetic disease prevention and control group, birth defect prevention and control committee, Chinese Preventive Medicine Association. (2019). Expert consensus on the application of low-depth whole genome sequencing technology in prenatal diagnosis. Chin. J. Med. Genet..

[13] Robson SC (2017). Evaluation of Array Comparative genomic Hybridisation in prenatal diagnosis of fetal anomalies: a multicentre cohort study with cost analysis and assessment of patient, health professional and commissioner preferences for array comparative genomic hybridisation. Efficacy Mechanism Evaluation.

[14] Wang J (2018). Prospective chromosome analysis of 3429 amniocentesis samples in China using copy number variation sequencing. Am. J. Obstet. Gynecol..

[15] Hillman SC (2011). Additional information from array comparative genomic hybridization technology over conventional karyotyping in prenatal diagnosis: a systematic review and meta-analysis. Ultrasound Obstet. Gynecol..

[16] Hillman SC (2013). Use of prenatal chromosomal microarray: prospective cohort study and systematic review and meta-analysis. Ultrasound Obstet. Gynecol..

[17] Wapner RJ (2012). Chromosomal microarray versus karyotyping for prenatal diagnosis. N. Engl. J. Med..

[18] Richards S (2015). ACMG Laboratory Quality Assurance Committee. Standards and guidelines for the interpretation of sequence variants: a joint consensus recommendation of the American College of Medical Genetics and Genomics and the Association for Molecular Pathology. Genet. Med..

[19] Zarocostas J (2006). Serious birth defects kill at least three million children a year. BMJ.

[20] Tabor A (1986). Randomised controlled trial of genetic amniocentesis in 4606 low-risk women. Lancet.

[21] Akolekar R, Beta J, Picciarelli G, Ogilvie C, D’Antonio F (2015). Procedure-related risk of miscarriage following amniocentesis and chorionic villus sampling: a systematic review and meta-analysis. Ultrasound Obstet. Gynecol..

[22] Wulff CB (2016). The risk of fetal loss associated with invasive testing following combined first trimester risk screening for Down syndrome – a national cohort of 147 987 singleton pregnancies. Ultrasound Obstet. Gynecol..

[23] ENCODE Project Consortium. (2012). An integrated encyclopedia of DNA elements in the human genome. Nature.

[24] Fu QH, Zheng ZJ (2013). Chromosomal microarray analysis in prenatal diagnosis. Chin. J. Lab. Med..

[25] Ghosh S (2016). Human RAD52: a novel player in DNA repair in cancer and immunodeficiency. Haematologica.

[26] Kuhlen M (2016). De novo PIK3R1 gain-of-function with recurrent sinopulmonary infections, long-lasting chronic CMV-lymphadenitis and microcephaly. Clin. Immunol..

[27] Zhu X (2015). Whole-exome sequencing in undiagnosed genetic diseases: interpreting 119 trios. Genet. Med..

